# Combining different selection criteria into breeding strategies for short-term recurrent genomic selection

**DOI:** 10.1007/s00122-026-05410-7

**Published:** 2026-10-25

**Authors:** Emanuel Grosch, Matthias Herrmann, Selma Schurack, Steffen Beuch, Vivek Kurra, Martin Mascher, Raz Avni, Klaus Oldach, Ina Röhrs, Wubishet Bekele, Anja Hanemann, Raja Ram Mehta, Carsten Reinbrecht, Albrecht Serfling, Andreas Stahl, Marco Stucke, Carola Zenke-Philippi

**Affiliations:** 1 Institute of Agronomy and Plant Breeding II, Justus Liebig University, Heinrich-Buff-Ring 26-32, 35392 Gießen, Germany; 2 Institute for Breeding Research on Agricultural Crops, Julius Kühn Institute, Federal Research Centre for Cultivated Plants, Rudolf-Schick-Platz 3a, 18190 Sanitz, Germany; 3 Saatzucht Granskevitz, Nordsaat Saatzucht GmbH, Granskevitz 3, 18569 Schaprode, Germany; 4 Saatzucht Bauer GmbH & Co. KG, Landshuter Str. 3a, 93083 Obertraubling, Germany; 5 Leibniz Institute of Plant Genetics and Crop Plant Research (IPK) Gatersleben, Corrensstraße 3, 06466 Seeland, Germany; 6 KWS Lochow GmbH, Ferdinand-von-Lochow-Str. 5, 29303 Bergen, Germany; 7 Research & Breeding Dottenfelderhof, Landbauschule Dottenfelderhof e.V., Dottenfelderhof 1, 61118 Bad Vilbel, Germany; 8 Ottawa Research and Development Centre, Agriculture and Agri-Food Canada, 960 Carling Avenue, K1A 0C6 Ottawa, Ontario, Canada; 9 Saatzucht Josef Breun GmbH & Co. KG, Amselweg 1, 91074 Herzogenaurach, Germany; 10 Saatzucht Streng-Engelen GmbH & Co. KG, Aspachhof, 97215 Uffenheim, Germany; 11 Institute for Resistance Research and Stress Tolerance, Julius Kühn Institute, Federal Research Centre for Cultivated Plants, Erwin-Baur-Str. 27, 06484 Quedlinburg, Germany

## Abstract

Each breeding program must find a balance between short-term genetic gain and the preservation of genetic variation. Different selection criteria, cross selection indices and cross-planning methods have been proposed in order to improve the trade-off between both goals compared to truncation selection and mating best with best. The relative advantage of each of these measures and methods may change depending on the duration of the breeding program because each has its particular strengths and weaknesses. We specifically focused on a short-term recurrent genomic selection program spanning five generations and used two datasets with 224 oat and 293 wheat lines, respectively, for a simulation study which evaluated genetic gain and genetic diversity for 19 different breeding strategies. Overall, we found that strategies that combined different selection criteria, cross selection indices, and/or cross-planning methods rather than relying on just one measure alone resulted in a higher genetic gain compared to truncation selection while maintaining at least the same amount of genetic diversity. We therefore conclude that combining different measures into breeding strategies can enhance breeding progress without any need for additional resources.

**Supplementary Information:**

The online version contains supplementary material available at https://doi.org/10.1007/s00122-026-05410-7.

## Key message

Combining different selection criteria, cross selection indices, and cross-planning methods into breeding strategies increases genetic gain in short-term recurrent genomic selection compared to truncation selection with random assignment of mates.

## Introduction

Genomic selection (GS) of quantitative traits raises the attention of many plant breeders due to lower costs and higher genetic gain than phenotypic selection (Muleta et al. 2019). However, truncation selection based on the genomic estimate of the genotypic value (GEGV), or short: genotypic value, showed, compared to phenotypic selection, a greater loss of diversity, hindering long-term success (Tessema et al. 2020). Extensive research has been conducted in the last two decades in order to find methods for genomic selection that find a better balance between short-term genetic gain and the preservation of genetic diversity.

In order to prevent the loss of favorable alleles, weighted GS was suggested as an alternative that places a higher weight on rare alleles with positive effects (Goddard 2009; Jannink 2010). Both the optimal haploid value (OHV) (Daetwyler et al. 2015) and the expected maximum haploid breeding value (EMBV) (Müller et al. 2018) focus on the gametes that are produced by the selection candidates. The OHV first divides the chromosomes into haplotype blocks and then measures the theoretical genomic estimate of the breeding value (GEBV) that could be produced from a gamete of an individual that combines the best haplotype block variants, regardless of the number of recombination events that would be required to actually obtain this gamete. The EMBV can be calculated for single markers and is the GEBV of the top inbred lines that can be produced from the gametes of an individual, considering the actual number of offspring and recombination events.

After selection, the selected individuals have to be assigned to mating partners. This can be done randomly or systematically. For example, it is possible to rank the selected individuals and cross the best with the second best, the third best with the fourth best, *etc. *Cockerham and Burrows (1980) proposed dividing the rank list of selected individuals into an upper and a lower half and assigning mating partners to each other from both parts of the list in order to put more emphasis on long-term selection gain.

As an alternative, selection of parents can be replaced by selection of crosses to be conducted so that selection of parents and mate allocation are done in one step. The usefulness criterion (UC) as a cross selection index was originally proposed by Schnell and Utz (1975) and can be interpreted as the expectation of the selected fraction of individuals from a population. It has gained attention since methods for the estimation of the segregation variance from marker effects and linkage disequilibrium (LD) have become available (Lehermeier et al. 2017; Osthushenrich et al. 2017) and has recently been extended to more than one generation (Niehoff et al. 2024). An alternative cross selection index that does not use the genetic segregation variance is the best progeny value (BPV) (Michel et al. 2022). It considers the expected mean value of a cross as well as the expected difference between this expectation and the theoretically best individual in the progeny.

Other approaches focus on determining an optimal set of selection candidates rather than on selecting genotypes or crosses independently from each other. Optimal contribution selection aims to maximize genetic gain, measured by the GEBV, while placing contraints on the level of inbreeding (Woolliams et al. 2015). The concept was extended to optimal cross selection by the allocation of mating partners (Gorjanc et al. 2018) and by the use of the usefulness criterion in place of the GEBV (Allier et al. 2019). The optimal population value does not take any diversity restrictions into account but measures the sum of the best haplotype block effects in a set of individuals and aims to find the set with the maximum sum (Goiffon et al. 2017). Since finding the optimal sets of crosses for each of theses methods is challenging, different software solutions have been developed in the past years for this purpose, including the R packages SimpleMating (Peixoto et al. 2025), and Genomic Mating (Akdemir et al. 2019; Akdemir and Sánchez 2016), the software AlphaMate (Gorjanc and Hickey 2018), and a look-ahead approach (Moeinizade et al. 2019). The user interfaces allow the specification of the desired objective of the optimization, restraints on relatedness or inbreeding, the number of crosses, number of offspring per cross, *etc.*

While retaining genetic diversity is needed for long-term success, short-term genetic gain is necessary to refinance the breeding program. Most of the selection criteria, cross selection indices and cross-planning methods mentioned above increase genetic gain in the long run compared to truncation selection but result in a lower genetic gain in the first generations. A two-part breeding strategy which splits a breeding program into a population improvement and a product development part was suggested as a way to balance out these two goals. While genetic diversity can be maintained in the population improvement part, the genetic gain can be increased in the product development part (Gaynor et al. 2017). Adaptive scoping follows the same rationale, with a different focus on genetic diversity and genetic gain over a pre-defined timeframe (Vanavermaete et al. 2022).

The high number of different selection criteria, cross selection indices, and cross-planning methods listed above illustrates how difficult it is to decide which methods to use in a particular breeding program because they all come with different advantages and disadvantages. In this simulation study, we specifically focused on a short-term recurrent genomic selection program. We combined different selection criteria, cross selection indices, and cross-planning methods into breeding strategies in order to better exploit the specific strengths of each of the measures and methods. Our objective was to compare different breeding strategies with our reference strategy (truncation selection for the GEGV with random assignment of mating pairs) in terms of selection gain and genetic diversity in two datasets in oat and wheat. First, we considered strategies using one single selection criterion or cross selection index in several generations. Second, we investigated strategies that used one single selection criterion or cross selection index aimed at preserving genetic diversity early in the breeding scheme and truncation selection afterwards. The purpose was to investigate the influence of each of these measures on selection gain and genetic diversity. Third, from these observations, we derived breeding strategies heuristically as combinations of different selection criteria, cross selection indices, and/or cross-planning methods, in order to obtain breeding strategies that result in a higher selection gain than the reference while maintaining at least the same amount of genetic diversity.

## Material and methods

### Datasets

The oat dataset consisted of 238 F$_7$ inbred lines originating from factorial crosses between 15 oat genotypes (Table 1). Field data was collected in 2021 (augmented design at one location), 2022 (randomized complete block design with two replications at five locations), and 2024 (two replications under organic conditions at two locations) in Germany. The yield was analysed with a two-stage analysis with mixed linear models (Endelman 2023). The inbred lines were genotyped with genotyping by sequencing. Reads were mapped against the genome of the oat variety “Sang” (Kamal et al. 2022). After excluding all markers with more than two alleles, more than 10 percent missing data, an expected heterozygosity of less than 0.1, and individuals with more than 10 percent missing markers, 224 genotypes (Table 1) and 12,825 markers were retained.

**Table 1:** Size *n* of the families of F$_7$inbred lines derived from the crosses of Parent 1 x Parent 2 for the oat dataset

Parent 2			Parent 1			
	Jaak	Keely	Odal	PGL228	Puhti	Zorro
	*n*					
Apollon	5		3		3	8
Armani		12			6	2
DCAs2PGL-253inH817					21	
Delfin	8	7	2		12	
HSH_PanFläti				19	21	
Max		7	15		9	18
Sy12/1					22	
Sy3512				4		
Symphony	2		7		4	6

The wheat dataset consisted of doubled-haploid (DH) lines from factorial crosses between the elite wheat lines Asory (SECOBRA Saatzucht), Informer (Saatzucht Breun), Kamerad (SECOBRA Saatzucht), LG Initial (Limagrain), and LG Mocca (Limagrain) and the resistance donor lines 20812.2.2, XX41, XX45, STb19/Lorikeet, and HTRI1410 (Table 2). In 2022/23, 369 DH lines were evaluated in augmented Latin squares or augmented randomized complete block designs (RCBDs) at six locations with one replication each. Because of seed limitations, 20 DH lines were evaluated in all locations, 64 in only one and the rest in between that range. After the first year of field trials, all DH lines and the parental lines were genotyped with the 25k wheat Infinium array (SGS Institut Fresenius TraitGenetics section, Gatersleben). Genotypes that were found to be selfings of the parents or heterozygotes as well as lines with by cleaning not removable husks, and genotypes with missing marker data were excluded from further evaluations. In the second year, the remaining 296 DH lines were evaluated in augmented RCBDs at the same six location as in the year before with two replications each. The yield was analysed with a two-stage analysis (Endelman 2023). After quality control, 293 genotypes (Table 2) and 16,478 markers were available for further analyses.

We used ridge regression best linear unbiased prediction (RR-BLUP) to estimate marker effects for both datasets. RR-BLUP was technically implemented using a transformation to an animal model (Shen et al. 2013). In order to obtain more robust results in case singular design matrices occur, we used method 2 of Nazarian and Gezan (2016).

**Table 2:** Size *n* of the families of DH lines derived from the crosses of parent 1 x parent 2 for the wheat dataset

Parent 2			Parent 1		
	20812.2.2	XX41	XX45	Stb19/Lorikeet	HTRI1410
	*n*				
Asory	15			16	38
Informer	1			16	9
Kamerad	29			21	1
LG Initial	23	9	4	41	11
LG Mocca	22			20	17

For both oat and wheat, the genetic map was approximated from the physical positions of the markers obtained in the empirical datasets assuming 1 Mbp = 1 cM since no suitable genetic maps were available.

Afterwards, the length of the chromosomes was scaled to an average length of 135 cM for oat and 180 cM for wheat.

### Breeding scheme of the recurrent genomic selection program and evaluation of the breeding strategies

**Fig. 1:**
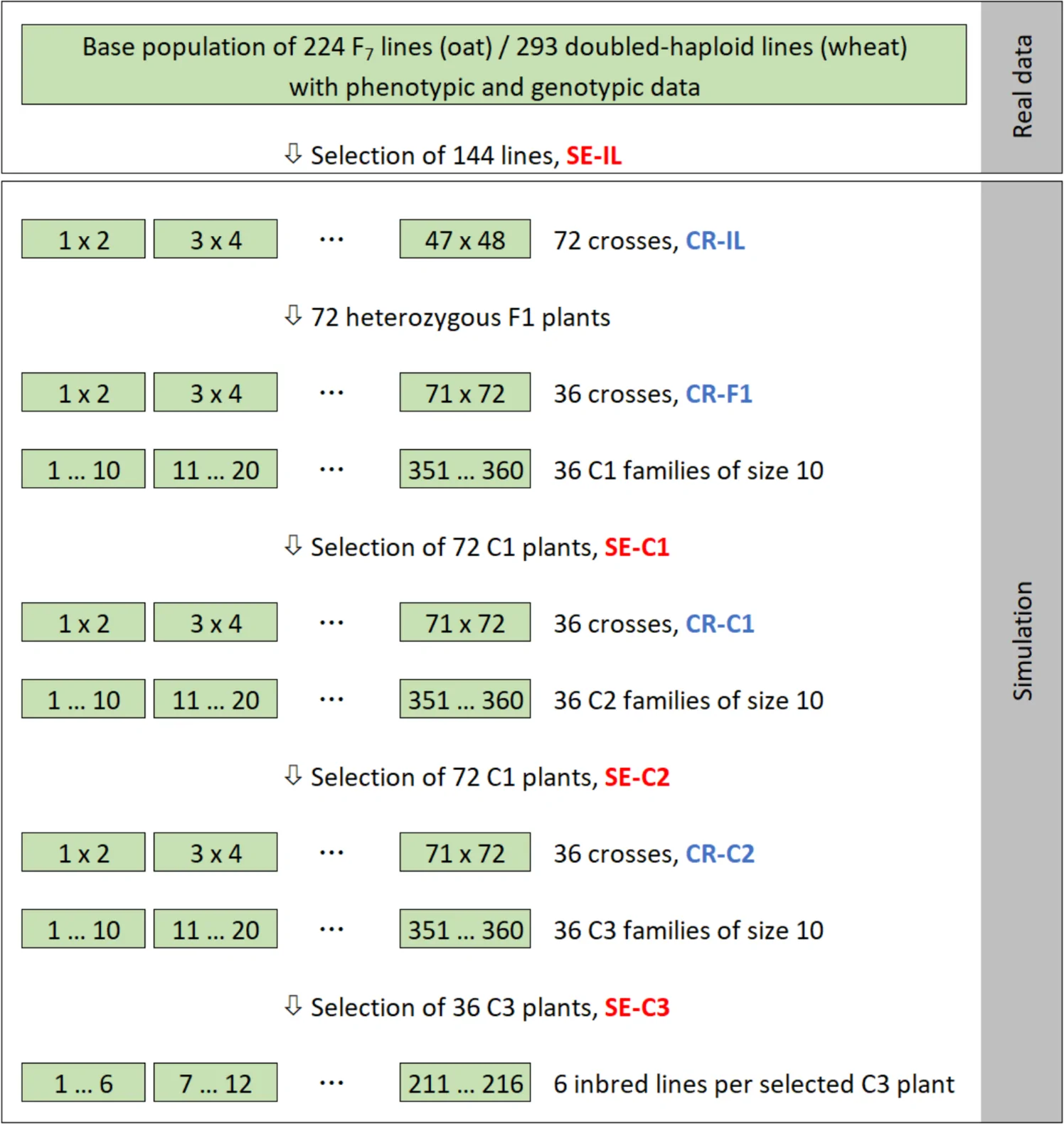
Breeding scheme for the simulated recurrent genomic selection program. Each selected plant was used in exactly one cross

The same breeding scheme (Fig. 1) was used for simulations in both datasets. In the first step, 144 parents out of the 224 (oat) or 293 (wheat) lines were selected by their GEGV (SE-IL). This step was based on real data. All following steps were simulated. Those 144 lines were used as the parents of the 72 F1 crosses (CR-IL), resulting in 72 F1 offspring. Each of the heterozygous F1 plants was crossed with one other F1 plant in cycle 1 (CR-F1) and 10 progeny were derived from each of these 36 crosses, resulting in 36 C1 families of size 10. The selection and crossing steps were repeated two more times (SE-C1/CR-C1 and SE-C2/CR-C2 in cycles 2 and 3, respectively) in order to create 360 plants from 36 C3 families of size 10. Of these, 36 were selected as donors for the final inbred lines (SE-C3) and 6 lines per donor were created, resulting in 216 inbred lines total. In oat, the final inbred lines were assumed to be obtained with single-seed descent with 5 selfing generations. In wheat, the final inbred lines were assumed to be DH lines. The selection of parents and assignment to mating partners was dependent on the breeding strategy.

The mean GEGV of the final 216 inbred lines as well as their mean genetic diversity, measured by the expected heterozygosity, were used to evaluate the different strategies in 300 simulation runs. The expected heterozygosity is the expected frequency of heterozygotes under Hardy-Weinberg equilibrium, given the observed allele frequencies from the simulated data. It ranges from 0 to 1 and is calculated as

$$\begin{aligned} \text {ExHet} = \frac{1}{n} \sum _{j = 1}^{n} (1 - p_j^2 - (1-p_j)^2) \end{aligned}$$

where $p_j$ is the frequency of allele *p* at locus *j* and *n* is the total number of loci. As an additional measure of diversity, we counted the number of fixed alleles in the DH generation. These can be divided into those with positive effects and those with negative effects for the selected trait.

### Selection criteria and cross selection indices

We define a selection criterion as the metric for selecting individuals *l* and a cross selection index as the metric for the ranking of potential crosses *q*.

***Genomic estimate of the genotypic value (GEGV)***

 The GEGV of an individual *l* is the sum of the additive effects $a_j$ of the *j* loci on the *c* chromosomes, estimated with RR-BLUP:

$$\begin{aligned} \text {GEGV}_l = \beta _0 + \sum _{c}\sum _{j} t_{jl} a_j \end{aligned}$$

$\beta _0$ is the common mean, and the covariate $t_{jl} =$ 2, 1, and 0, for SNP genotypes $A_1 A_1$, $A_1 A_2$, and $A_2 A_2$, respectively.

***Expected maximum haploid genotypic value (EMGV)***

The expected maximum haploid breeding value (EMBV) of an individual *l* is the expected performance of the best among a limited number of its progeny (Müller et al. 2018). The EMGV presented here is not a breeding value but rather a genotypic value since additive effects and not allelic substitution effects were used for the simulations. It was obtained by simulating 10 DH lines per selection candidate, evaluating them by their GEGV and calculating the mean GEGV of the two best DH lines. We repeated this process 20 times and took the mean of all replications as the EMGV of each genotype.

***Best progeny value (BPV)***

The BPV of a cross *q* was calculated as

$$\begin{aligned} \text {BPV}_{q} = \mu + E(\Delta _{\text {max}}) \end{aligned}$$

where $E(\Delta _{\text {max}})$ can be expressed as

$$\begin{aligned} E(\Delta _{\text {max}}) = \sum _{j} \left[ 1 - ( p_j - (1-p_j) ) ^2 \right] |a_j| \end{aligned}$$

(Michel et al. 2022). In homozygous offspring, $E(\Delta _{\text {max}})$ is the expected difference between *μ* and the genotypic value of the theoretically best offspring that carries favourable alleles at all segregating loci. The term that is used to weight the absolute additive effect is twice the expected heterozygosity at a single locus.

***Maximum genomic estimate of the genotypic value (MAXG)***

The cross selection index MAXG is the maximum genetic value of a DH line that could be expected if that individual received the best alleles for all genes from its parents. It considers the best of the four additive effects of the two alleles of the first parent, $g_j$, $g_j^\prime$, and of the second parent, $h_j$, $h_j^\prime$, in a cross *q* on all loci *j* of a chromosome, and sums over chromosomes *c*:

$$\begin{aligned} \text {MAXG}_{q} = \beta _0 + \sum _{c}\sum _{j} max(g_j, g_j^\prime ,h_j, h_j^\prime ) \end{aligned}$$

MAXG is related to the optimal haploid value (OHV) (Daetwyler et al. 2015) but uses additive effects for single markers rather than allelic substitution effects and haplotype blocks. For a cross between two fully homozygous parents, $BPV_{q} = MAXG_{q}$.

***Usefulness criterion (UC)***

The usefulness criterion is calculated by the mean *μ* and the genetic standard deviation $\sigma _g$ of the progeny derived from a cross between two individuals:

$$\begin{aligned} \text {UC}_{q} = \mu + i \sigma _g \end{aligned}$$

It is the usefulness (Schnell and Utz 1975) when assuming that the heritability of marker scores is one, which was termed superior progeny value (Zhong and Jannink 2007), and can be derived analytically from marker effects and recombination frequencies between loci (Lehermeier et al. 2017). The relative importance given to the genetic variance depends on the selection intensity *i*. More emphasis is placed on $\sigma _g$ when the selection intensity is high, *i.e.* only a small fraction of all individuals is selected. The selection intensity was chosen according to our breeding scheme as *i = 1.4* which corresponds to selecting 72 out of 360 plants.

The means and standard deviations for a population of heterozygotes derived from two heterozygous parents can be expressed in terms of the additive effects $g_j$, $g_j^\prime$ of the first and $h_j$, $h_j^\prime$ of the second parent and the recombination frequencies $r_{jk}$ between two loci *j* and *k*. The derivation can be found in Supplementary File 1. It is in analogy to the derivations of Osthushenrich et al. (2017) and is mathematically equivalent to the formulations presented by Bonk et al. (2016).

***Expected genotypic value of grand offspring (ExGrO)***

The cross selection index ExGrO is based on the metric ExpBVSelGrOff from Niehoff et al. (2024) which is the breeding value of selected grand-offspring produced by selected offspring of a cross *q*. It is the sum of the selection differential $\mu + i \sigma _g$ realized in the offspring and the selection differential realized in the grand-offspring of a particular cross *q* between two parents *l* and *m* and was calculated with the formulas presented in Niehoff et al. (2024) as:

$$\begin{aligned} \text {ExGrO}_{q} = 0.25 (\text {GEGV}_{l} + \text {GEGV}_{m}) + i (0.5 \sqrt{\sigma ^{2}_{FS}} \sqrt{\sigma ^{2}_{GO}}) \end{aligned}$$

where the selection intensity *i* is 1.4, $\sqrt{\sigma ^{2}_{FS}}$ is the genetic variance of the full-sib offspring of cross *q*, and $\sqrt{\sigma ^{2}_{GO}}$ is the genetic variance of grand-offspring produced by selected offspring cross *q* when mated to a random partner of selected individuals of the population from the same generation. Again, ExGrO is a genotypic value rather than a breeding value because additive effects were used.

***Genetic distance (GDI)***

The genetic Rogers’ distance (Rogers 1972) for all possible cross combinations was used as the cross selection index GDI.

### Cross-planning methods

We define a cross-planning method as the procedure which either assigns selected parents to the crosses (for selection criteria GEGV and EMGV) or selects crosses from a list of all potential crosses that have been evaluated with a particular cross selection index (for BPV, MAXG, UC, and ExGrO). We did not use the software AlphaMate (Gorjanc and Hickey 2018) because with just one cross per parent, as implemented in our breeding scheme (Fig. 1), the result was similar to truncation selection for the GEGV with random mating. Similar, the look-ahead approach by Moeinizade et al. (2019) was not applicable to our breeding scheme with equal contributions of parents. With Genomic Mating as suggested in Akdemir and Sánchez (2016) and available from https://github.com/cran/GenomicMating, we were not able to obtain any solution for the cross-planning method.

***Cross-planning methods for assigning selected parents to crosses***

 In cross-planning method RND, the selected parents were assigned randomly to the crosses. In cross-planning method BEST, all selected parents were listed by their selection criterion in decreasing order. Then, the first genotype on the list was assigned to the second as its mating partner, the third to the fourth, *etc.*. In cross-planning method CB80, the superior half of the selected fraction was crossed with the inferior half (Cockerham and Burrows 1980). In our implementation we ordered the genotypes by their GEGV and split the list into an upper and a lower half. Now, the genotype on the first position in the upper half was crossed with the genotype on the first position in the lower half, *etc.*

***Cross-planning methods for selecting crosses to be conducted***

In cross-planning method TOP, we ranked the potential crosses in descending order according to their cross selection index, selected the cross with the greatest value and removed all combinations with one of the two parents from the list. In the next step, we again selected the cross with the greatest value for the cross selection index from the remaining crosses on the list and repeated this process until the desired number of crosses was reached.

Cross-planning method SET was inspired by optimal cross selection (Allier et al. 2019) and optimizes the overall value of the cross selection index for a set of crosses heuristically, in analogy to Goiffon et al. (2017). We repeated the procedure in cross-planning method TOP 200 times. After each repetition, we removed the very first cross on the ordered list before we started again. This way, we ended up with 200 different sets of suitable crosses. We then calculated the mean value for the cross selection index for each of the 200 sets and carried out the crosses in the set with the greatest mean value.

In cross-planning method SM, we used the R package SimpleMating (Peixoto et al. 2025) which maximizes the selection criterion or cross selection index with an optimization algorithm under a threshold for relatedness. We calculated the genomic relationship matrices $\textbf{G}$ between the parents according to VanRaden (2008). Then we used SimpleMating to obtain the crossing plan with the lowest possible values for the relatedness threshold (1.3 for oat and 0.5 for wheat) that did not result in an error.

For cross-planning method ExGrO, we found suitable cross combinations by using the algorithm for selection of parents proposed by Niehoff et al. (2024) and then randomly assigning the selected parents to the crosses.

### Breeding strategies

Simulations were carried out for 19 different breeding strategies (Table 3). Each breeding strategy was a unique combination of selection criteria/cross selection indices and cross-planning methods at different stages of the recurrent genomic selection program. Strategy GEGV-RND as an established breeding strategy was used as a reference. In this strategy, all selection steps were based on the GEGV and selected parents were mated randomly. All other breeding strategies differed from strategy GEGV-RND in one or more steps. They used one selection criterion/cross selection index throughout (strategies GEGV-BEST, EMGV-RND, BPV-TOP, MAXG-TOP, UC-SM, UC-SET, and EXGRO), were based on a selection criterion/cross selection index or cross-planning method that aimed to maximize diversity early in the breeding program (strategies GEGV-CB80, GEGV-GDI_TOP, MAXG1-TOP, and MAXG2-TOP), or combined different cross selection indices and/or cross-planning methods (strategies MAXG1/UC1-TOP, MAXG2/UC1-TOP, MAXG2-TOP/UC1-SM, MAXG2-TOP/UC1-SET, MAXG2/UC2-TOP, MAXG2/BPV2-TOP, and MAXG/BPV_ALT-TOP).

**Table 3:**
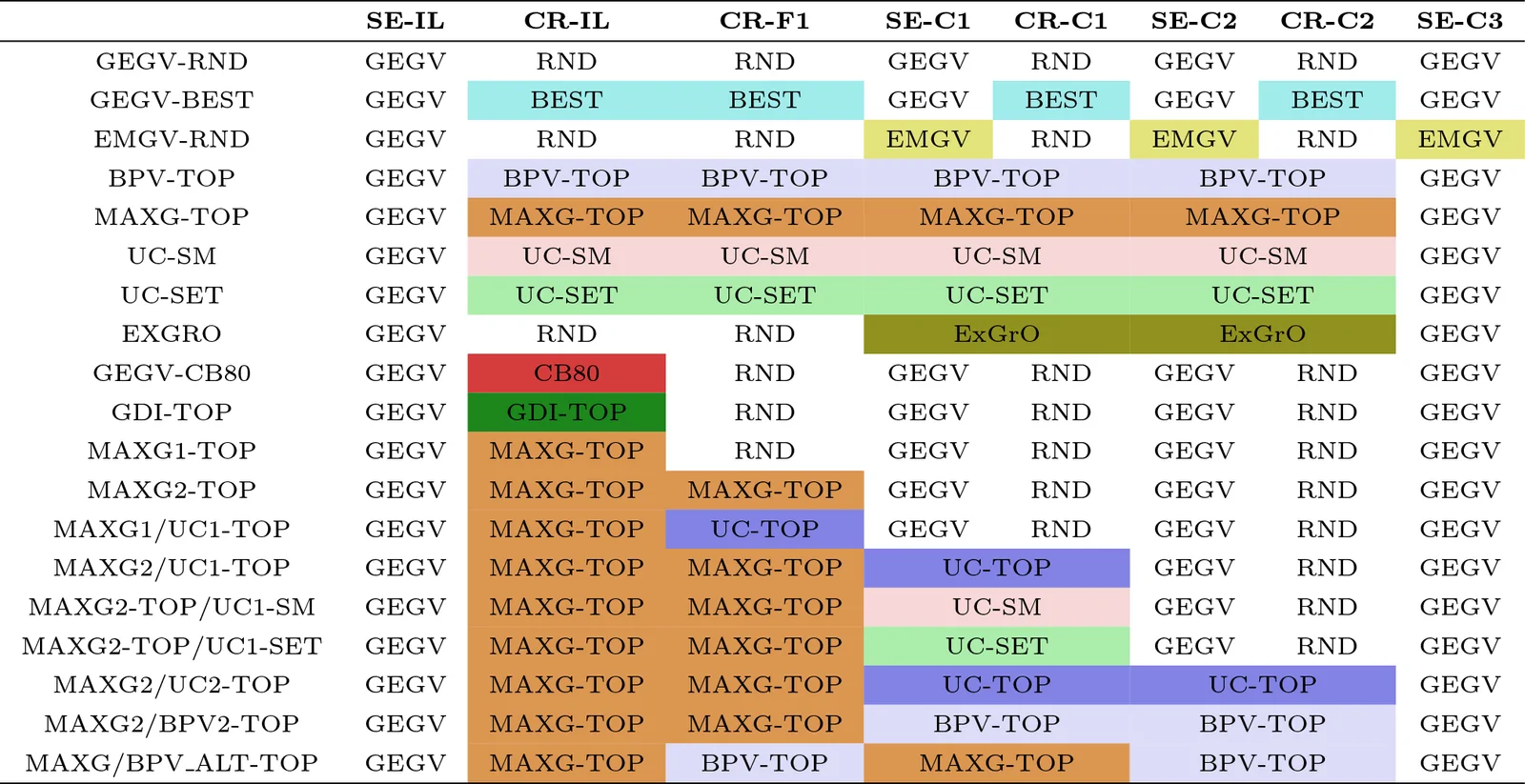
Overview over the simulated breeding strategies. SE: selection, CR: assignment of mating partners to crosses

### Software

All calculations were performed with R version 4.5.0 (R Core Team 2025) on an Intel Xeon Gold 6330 CPU @ 2.00GHz. The yield trial evaluations were carried out with the R-package StageWise, version 1.15 (Endelman 2023) and ASReml-R version 4.2 (The VSNi Team 2023). The simulations were carried out with customized routines in the C and R programming languages which were based on the simulation routines of the software Plabsoft (Maurer et al. 2008). Estimation of marker effects with RR-BLUP was carried out with customized C and R routines. The R package SimpleMating, version 0.0.9002 (Peixoto et al. 2025), was used for obtaining the crossing plan for cross-planning method SM. We used the R package AGHmatrix, version 2.1.4 (Amadeu et al. 2023), to calculate the genomic relationship matrices needed for cross-planning method SM. Results were visualized with R package ggplot2, version 3.5.2.

## Results

**Fig. 2:**
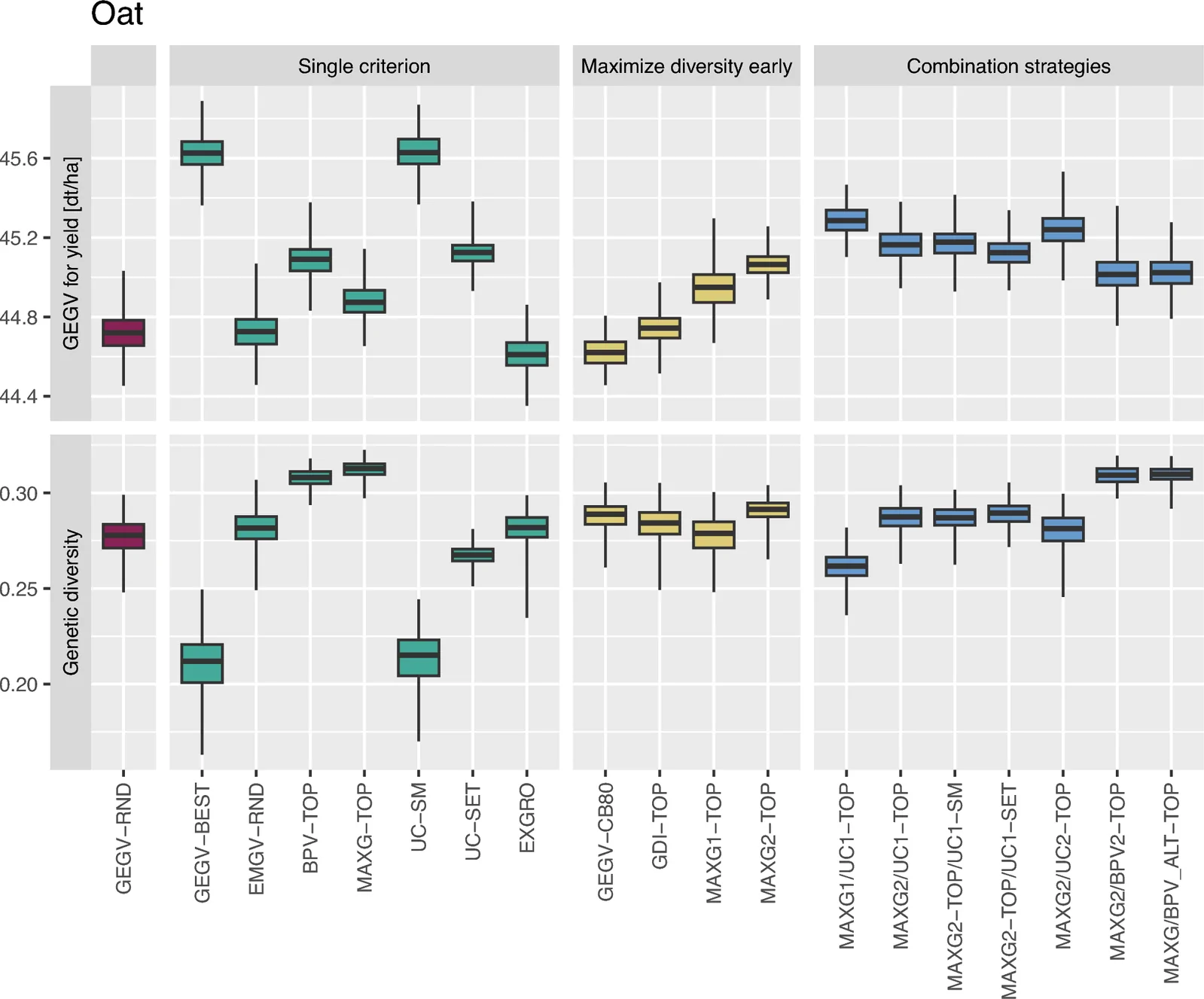
Genomic estimates of the genotypic values (GEGVs) for yield and genetic diversity, measured by the expected heterozygosity, for different breeding strategies in the oat dataset. Each boxplot is composed of the mean values for each measure in 300 simulation runs. The first boxplot shows the reference strategy (truncation selection for GEGV with random assignment of the selected parents to crosses)

**Fig. 3:**
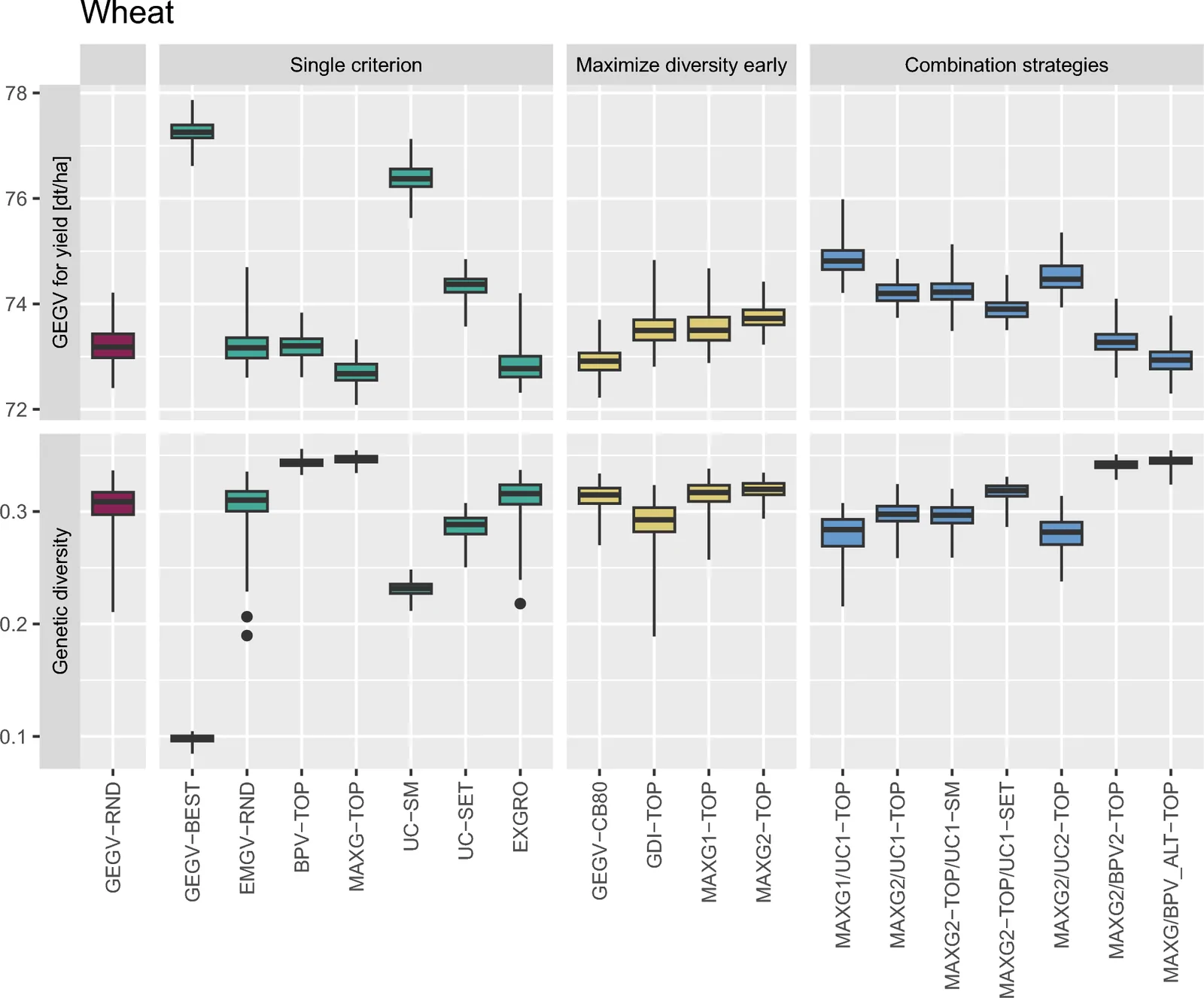
Genomic estimates of the genotypic values (GEGVs) for yield and genetic diversity, measured by the expected heterozygosity, for different breeding strategies in the wheat dataset. Each boxplot is composed of the mean values for each measure in 300 simulation runs. The first boxplot shows the reference strategy (truncation selection for GEGV with random assignment of the selected parents to crosses)

The results of the simulation study are visualized as the mean GEGV and the mean genetic diversity of the final inbred lines in Figs. 2 and 3. The corresponding numerical values for means and standard deviations can be found in Table 4.

Table 5 summarizes the average number of fixed alleles with positive and negative effects. The expected heterozygosity and the numbers of fixed alleles are negatively correlated, so they are not reported and discussed separately but rather broadly referred to as “genetic diversity”. The reference strategy is GEGV-RND, with truncation selection for the GEGV and random assignment of mating partners.

### Breeding strategies based on a single selection criterion or cross selection index

Overall, differences between the strategies were greatest in this group. None of the strategies led to a higher genetic gain while preserving more genetic diversity than GEGV-RND at the same time. Strategy GEGV-BEST, which used truncation selection for the GEGV and mating best with best, led to the highest selection gain and the lowest genetic diversity in both data sets. For strategies EMGV-RND and EXGRO, selection gain and genetic diversity were comparable to those of the reference strategy GEGV-RND. Strategy BPV-TOP resulted in a selection gain higher than GEGV-RND in oat and comparable to GEGV-RND in wheat while the genetic diversity was higher in both datasets. MAXG-TOP had a slightly higher (oat) or lower (wheat) selection gain than GEGV-RND and retained more diversity. The usefulness-based strategies UC-SM and UC-SET resulted in a higher genetic gain and a lower genetic diversity than GEGV-RND. The differences were more pronounced for UC-SM.

### Breeding strategies that aim to maximize genetic diversity early in the breeding scheme

In both datasets, the genetic gain with GEGV-CB80 was lower than with GEGV-RND while the genetic diversity was comparable (wheat) or slightly higher (oat). For strategy GDI-TOP, genetic gain was higher and genetic diversity lower in wheat. In oat, genetic gain was equal to that of GEGV-RND and the genetic diversity higher. A higher genetic gain compared to the reference was observed with strategy MAXG1-TOP while the genetic diversity was comparable to that of GEGV-RND (oat) or slightly higher (wheat). Strategy MAXG2-TOP resulted in a higher genetic gain and a higher genetic diversity than GEGV-RND and also than MAXG1-TOP in both datasets.

### Breeding strategies that combine different cross selection indices and/or cross-planning methods

Genetic gain was higher than with GEGV-RND in all investigated combination strategies. The inverse correlation between genetic gain and genetic diversity was most pronounced in this group. For breeding strategy MAXG1/UC1-TOP, we observed the highest genetic gain of all combination strategies but the lowest genetic diversity, lower than with GEGV-RND. With MAXG2/UC1-TOP, the genetic gain was higher than with GEGV-RND but lower than with MAXG1/UC-TOP. while diversity was higher in oat and lower in wheat. The results with MAXG2-TOP/UC1-SM were the same as for MAXG2-UC1-TOP. For strategy MAXG2-TOP/UC1-SET, both genetic gain and genetic diversity were greater than with GEGV-RND. For strategy MAXG2/UC2-TOP, the genetic diversity was comparable to that obtained with GEGV-RND in oat and lower in wheat. The strategies that combine MAXG with BPV, either consecutively (MAXG2-BPV2-TOP) or alternating (MAXG/BPV_ALT-TOP), retained the most genetic diversity while being those with the smallest genetic gain of all combination strategies. In wheat, the genetic gain with MAXG2-BPV2-TOP was higher than with MAXG/BPV_ALT-TOP.

**Table 4:** Mean and standard deviation of genetic gain and genetic diversity for the tested strategies in oat and wheat. Different letters indicate significant differences on a 5 % significance level with false discovery rate adjustment. Since the power of significance tests depends on the sample size, the presented significances are specific to the scenario presented here which uses 300 replications

Strategy	Oat		Wheat	
	Genetic gain	Genetic diversity	Genetic gain	Genetic diversity
GEGV-RND	44.72 ± 0.09 ^b^	0.277 ± 0.009 ^e^	73.21 ± 0.31 ^d^	0.305 ± 0.018 ^g^
GEGV-BEST	45.63 ± 0.09 ^m^	0.210 ± 0.015 ^a^	77.27 ± 0.20 ^n^	0.098 ± 0.004 ^a^
EMGV-RND	44.73 ± 0.10 ^b^	0.281 ± 0.009 ^f^	73.21 ± 0.33 ^d^	0.307 ± 0.018 ^g^
BPV-TOP	45.09 ± 0.09 ^h^	0.308 ± 0.004 ^l^	73.19 ± 0.22 ^d^	0.343 ± 0.004 ^jk^
MAXG-TOP	44.88 ± 0.09 ^d^	0.312 ± 0.004 ^n^	72.70 ± 0.22 ^a^	0.346 ± 0.004 ^l^
UC-SM	45.63 ± 0.09 ^m^	0.213 ± 0.015 ^b^	76.42 ± 0.23 ^m^	0.231 ± 0.007 ^b^
UC-SET	45.12 ± 0.06 ^i^	0.267 ± 0.005 ^d^	74.33 ± 0.21 ^j^	0.287 ± 0.011 ^d^
EXGRO	44.61 ± 0.09 ^a^	0.281 ± 0.008 ^f^	72.84 ± 0.31 ^b^	0.313 ± 0.017 ^h^
GEGV-CB80	44.62 ± 0.07 ^a^	0.288 ± 0.007 ^ij^	72.92 ± 0.25 ^c^	0.313 ± 0.012 ^h^
GDI-TOP	44.75 ± 0.08 ^c^	0.284 ± 0.009 ^g^	73.54 ± 0.33 ^f^	0.289 ± 0.022 ^e^
MAXG1-TOP	44.95 ± 0.11 ^e^	0.278 ± 0.010 ^e^	73.55 ± 0.34 ^f^	0.315 ± 0.013 ^h^
MAXG2-TOP	45.07 ± 0.06 ^g^	0.291 ± 0.006 ^k^	73.74 ± 0.21 ^g^	0.319 ± 0.008 ^i^
MAXG1/UC1-TOP	45.29 ± 0.07 ^h^	0.262 ± 0.008 ^c^	74.85 ± 0.30 ^l^	0.279 ± 0.019 ^c^
MAXG2/UC1-TOP	45.17 ± 0.08 ^j^	0.287 ± 0.007 ^hi^	74.21 ± 0.22 ^i^	0.297 ± 0.011 ^f^
MAXG2-TOP/UC1-SM	45.17 ± 0.08 ^j^	0.287 ± 0.008 ^h^	74.25 ± 0.25 ^i^	0.296 ± 0.010 ^f^
MAXG2-TOP/UC1-SET	45.12 ± 0.07 ^i^	0.289 ± 0.006 ^j^	73.90 ± 0.19 ^h^	0.318 ± 0.007 ^i^
MAXG2/UC2-TOP	45.24 ± 0.08 ^k^	0.280 ± 0.009 ^f^	74.51 ± 0.27 ^k^	0.280 ± 0.015 ^c^
MAXG2/BPV2-TOP	45.01 ± 0.09 ^f^	0.309 ± 0.005 ^lm^	73.29 ± 0.23 ^e^	0.342 ± 0.004 ^j^
MAXG/BPV_ALT-TOP	45.02 ± 0.08 ^f^	0.310 ± 0.004 ^m^	72.93 ± 0.23 ^c^	0.345 ± 0.004 ^kl^

**Table 5:** Number of fixed markers with positive and negative effect, as well as the total number of fixed markers in oat and wheat. The total numbers of markers were 12,285 in oat and 16,478 in wheat, respectively

Strategy	Fixed markers in oat			Fixed markers in wheat		
	Negative	Positive	Total	Negative	Positive	Total
GEGV-RND	408	554	963	292	746	1039
GEGV-BEST	1772	2395	4167	4449	7828	12277
EMGV-RND	347	509	856	274	716	990
BPV-TOP	292	485	777	59	289	348
MAXG-TOP	265	488	753	51	282	333
UC-SM	1723	2312	4035	1802	3295	5097
UC-SET	961	953	1914	372	978	1350
EXGRO	307	453	760	220	600	820
GEGV-CB80	224	402	626	211	638	849
GDI-TOP	343	499	842	366	908	1274
MAXG1-TOP	449	727	1176	193	644	836
MAXG2-TOP	338	649	986	167	629	795
MAXG1/UC1-TOP	821	1302	2123	397	1225	1621
MAXG2/UC1-TOP	405	800	1205	422	1223	1644
MAXG2-TOP/UC1-SM	402	797	1199	455	1283	1738
MAXG2-TOP/UC1-SET	367	726	1093	235	794	1029
MAXG2/UC2-TOP	478	931	1409	705	1804	2509
MAXG2/BPV2-TOP	284	493	777	68	341	409
MAXG/BPV_ALT-TOP	281	496	778	57	290	347

## Discussion

In breeding, there is always a trade-off between genetic gain and genetic diversity. To our knowledge, there is no study comparing several breeding strategies specifically for their short-term response in order to produce DH or inbred lines within three cycles of recurrent genomic selection. We used truncation selection and random assignment of mating partners (GEGV-RND) as the reference strategy of our simulations because it is simple to implement and understand and is used in both plant and animal breeding. Genetic gain and genetic diversity (expected heterozygosity and number of fixed alleles) were used as evaluation criteria. It is important to note that the meaning of genetic diversity changes in the context of a short-term breeding program, from providing a reservoir for future genetic gains to a measure for the variability in the final product of the selection, the DH or inbred lines. This variability allows for the selection for other important agronomic and resistance traits apart from yield.

### Breeding strategies based on a single selection criterion/cross selection index and cross-planning method

In a first step, we investigated breeding strategies that rely on a single selection criterion/cross selection index and cross-planning method alone. None of these strategies showed both a higher selection gain and a greater genetic diversity than the reference strategy GEGV-RND at the same time (Figs. 2 and 3, Tables 4 and 5).

Strategy GEGV-BEST differed from GEGV-RND in the assignment of mating partners: The genotype with the greatest GEGV was crossed to the one with the second-greatest, *etc.*. Consequently, the selection gain for this strategy was the greatest of all strategies, while the genetic diversity was lowest. Strategies EMGV-RND, employing the expected maximum haploid genotypic value (EMGV) (Müller et al. 2018) for selection of parents, and EXGRO, using the expected breeding value of selected grand progeny (Niehoff et al. 2024), show levels of genetic gain and genetic diversity comparable to those of GEGV-RND. This is in line with previous findings in which selection with EMBV and the expected breeding value of selected grand progeny was superior to selection with the GEGV only after five or more generations (Müller et al. 2018; Niehoff et al. 2024).

For strategy BPV-TOP, the genetic gain was higher than for GEGV-RND in oat and comparable in wheat, while the genetic diversity was higher in both cases. This is in accordance with the results from Michel et al. (2022) who introduced the BPV metric and attribute the difference to GEGV-RND to the number of alleles that are fixed and lost, respectively. Strategy MAXG-TOP resulted in a genetic gain that was slightly higher than for reference strategy GEGV-RND in oat and slightly lower in wheat. The observed genetic diversity was the highest across all strategies. MAXG is a measure for the GEGV of the best possible DH line produced from a cross of two parents, assuming that the line inherits the best allele at each locus. Since allele frequencies are ignored and the number of recombination events is limited in a breeding program, MAXG measures the potential of a cross to produce a superior DH line without considering the actual probability for this event. It is a theoretical value rather than an actual target for DH production because a DH line for which the GEGV approaches the MAXG can never be obtained. Its main advantage is that it is not computationally demanding since it is only twice the sum of the best alleles from two parents at each locus, that no knowledge about the allelic phase or even the map position is required, and that no partitioning of the chromosomes into haplotype blocks is necessary.

**Fig. 4:**
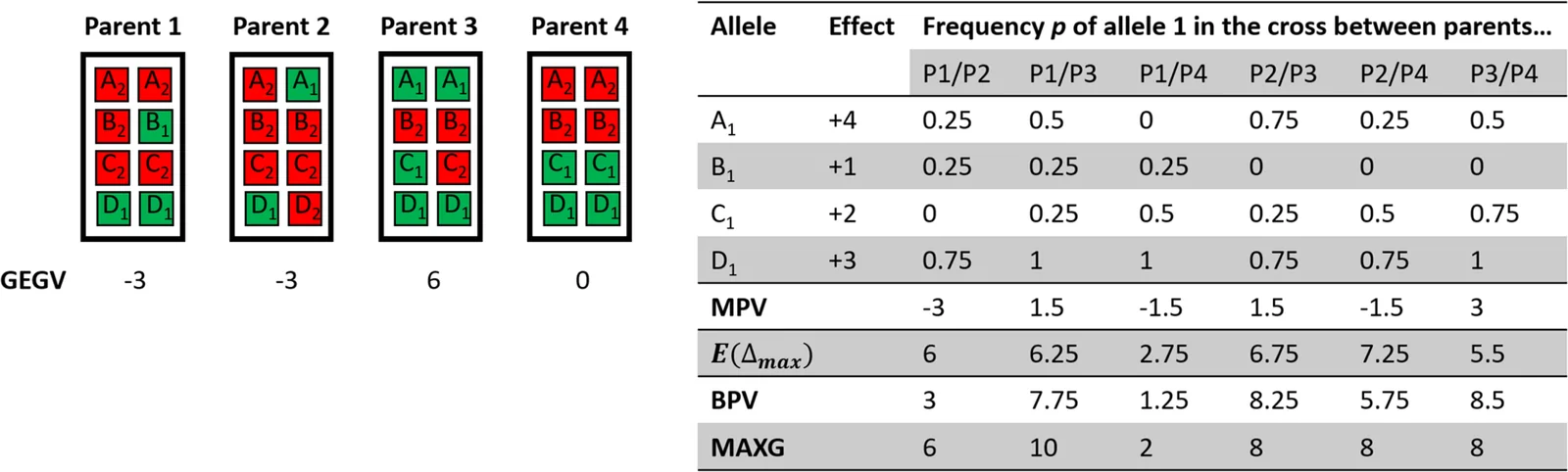
Example for selection with genomic estimate of the genotypic value (GEGV), mid-parent value (MPV), best progeny value (BPV), and maximum genomic estimate of the genotypic value (MAXG). The table states the effect of the reference allele. The allele frequencies are needed for the calculation of the BPV

**Fig. 5:**
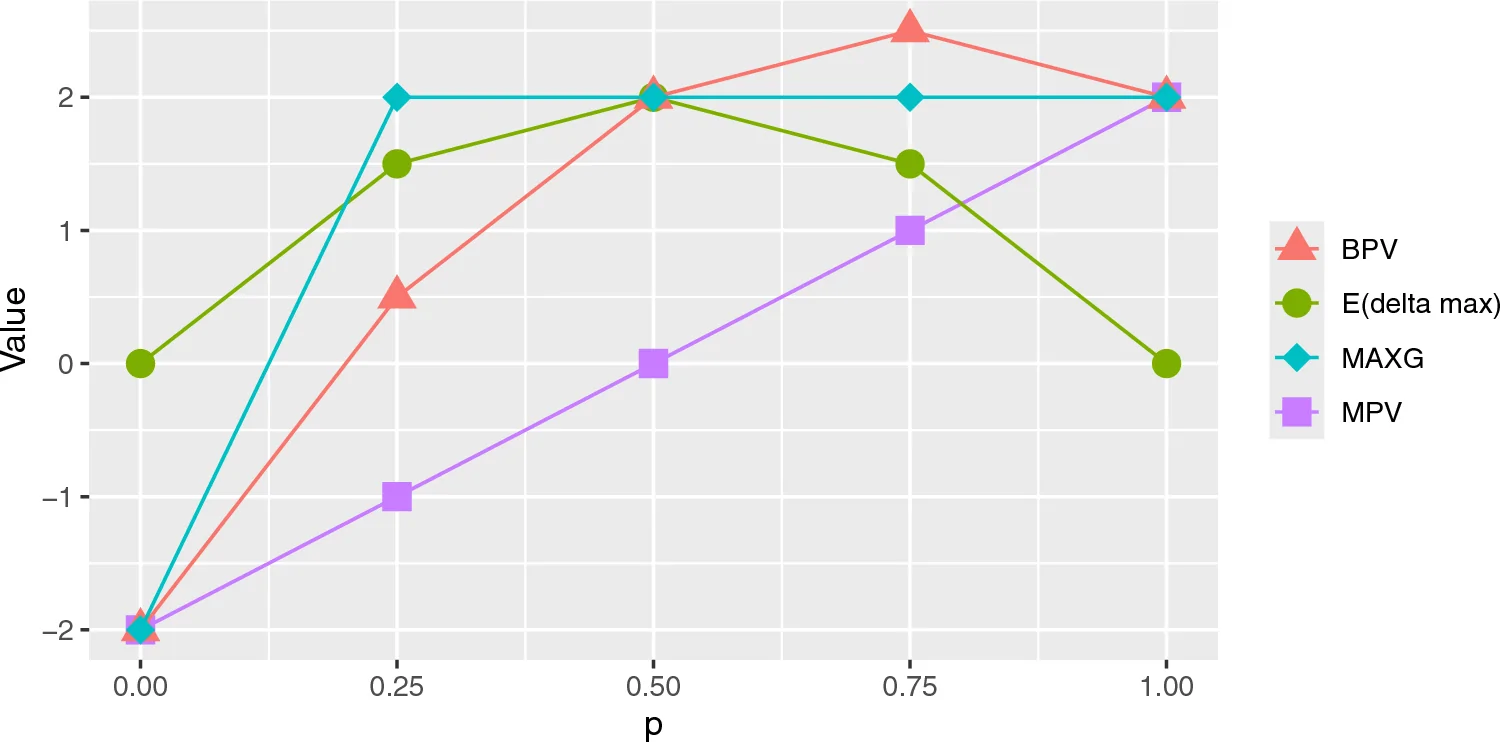
Values for best progeny value (BPV) which is the sum of the mid-parent value (MPV) and $E(\Delta _{\text {max}})$, and maximum genomic estimate of the genotypic value (MAXG) for different frequencies *p* of the reference allele $\text {A}_1$ with effect +2 in a cross between to parents. Only a single locus is considered

Figure 4 illustrates the different behaviours of GEGV, BPV, and MAXG. Suppose that the goal is to find the best possible cross between parents 1 through 4. With the GEGV, parents 3 and 4 would be selected and crossed. The BPV also identifies the cross between parents 3 and 4 as the best one. The expected mean value of this cross would be 3. With MAXG, the cross between parents 1 and 3, with an expected mean value of 1.5, would be selected. MAXG therefore leads to the selection of a cross that has a lower expected mean value, *i.e.* a smaller immediate selection gain, but preserves the favorable allele 1 of locus B. BPV assigns a lower value to this allele because of its low frequency and its relatively small effect compared to the positive alleles at the other loci while for MAXG, the allele frequencies and thereby the actual probabilities for a good allele to actually show up in the offspring are ignored. Figure 5 clarifies this point, considering only a single locus in a cross between two parents. While MAXG has the same value for all frequencies of the reference allele *p* with the exception of *p=0*, the BPV is greatest for *p=0.75*. The BPV therefore balances a high mid-parent value while rewarding heterozygosity, favoring a high frequency, but not fixation of the positive allele. This is in accordance with our simulation results: MAXG retains more genetic diversity at the cost of genetic gain.

Strategies UC-SM and UC-SET, both based on the usefulness criterion with different algorithms for obtaining the crossing plan, showed a higher genetic gain but a lower genetic diversity than GEGV-RND. This effect was stronger for strategy UC-SM (Peixoto et al. 2025) than for UC-SET, despite the fact that UC-SM uses an optimization algorithm from the R package SimpleMating which includes a relatedness threshold to ensure a minimal relatedness between mating partners. Relatedness restrictions in UC-SM were set as strictly as possible with the genomic relationship matrix $\textbf{G}$ but it is possible that they were not low enough to have an impact on the preservation of genetic diversity. We did not use any diversity threshold in strategy UC-SET. Our results seem to contradict other simulations that show only modestly higher genetic gains and slightly lower genetic diversity for the usefulness criterion and optimal cross selection compared to GEGV-RND in the first few generations of selection (Peixoto et al. 2025), even in the absence of any restraint on diversity (Allier et al. 2019). We attribute these differences to the different setup of our breeding scheme, namely the fact that each selected parent is only used in one cross and that only 10 offspring per cross are produced. This increases the chances that, in order to obtain a high genetic gain, the genetic backgrounds from single parents are over-represented in the selected fraction. Additionally, even though the inclusion of the segregation variance should theoretically lead to the preservation of rare alleles, in reality, they are probably lost because of Mendelian sampling due to the small family sizes. Using cross-planning method SET, which aims to find an optimal set of mating partners rather than starting with the cross with the highest UC, *etc.*, seems to reduce this effect and increases the chances of more balanced contributions of the families to the next generation.

### Breeding strategies that aim to maximize genetic diversity early in the breeding program

Strategies GEGV-CB80, GDI-TOP, MAXG1-TOP and MAXG2-TOP are all based on the assumption that even in a short-term recurrent genomic selection breeding program, genetic diversity that is retained in the first generation(s) can be exploited later. While the mating partners are selected based on their GEGV (truncation selection), mating is not random but follows different rules for each method. They all differ from GEGV-RND in the cross-planning method used in generation IL (GEGV-CB80, GDI-TOP and MAXG1-TOP) or additionally in generation F1 (MAXG2-TOP).

GEGV-CB80 aims to increase the diversity in the F1 generation compared to RND by crossing the best 50% of the selected fraction with the lower 50% of the selected fraction (Cockerham and Burrows 1980). In our simulation the genetic gain was slightly lower than in GEGV-RND while the genetic diversity was slightly higher (Figs. 2 and 3). By partitioning the selected fraction into a upper and a lower part, matings between the best or the worst genotypes are avoided. The GEGVs of the F1 crosses were therefore more similar than in strategy GEGV-RND (results not shown). This increases the probability for selecting progeny from each family for the C1 generation, resulting in more similar variances for the C1 families and, in consequence, lower means of the selected fractions which ultimately reduces genetic gain in the final generation of our breeding scheme. While the split of the selected genotypes into an upper and a lower half was originally suggested in order to increase the genetic gain in the long run (Cockerham and Burrows 1980), we doubt that this can be realized because the level of genetic diversity that was retained with strategy GEGV-CB80 was comparable to and not higher than for strategy GEGV-RND (Figs. 2 and 3).

Strategy GDI-TOP assigns selected genotypes to their mating partners based on a high genetic distance. In our simulation, depending on the dataset, the genetic gain was higher or equal to the one in GEGV-RND. In oat, genetic gain was approximately equal to GEGV-RND but the diversity was higher. In wheat, the genetic gain was higher and the diversity was lower compared to strategy GEGV-RND (Figs. 2 and 3). Using the genetic distance between the founder lines in a breeding scheme in order to retain genetic diversity can therefore not be recommended.

Our results show that planning crosses based on MAXG early in the breeding scheme, as in strategies MAXG1-TOP and MAXG2-TOP, results in a higher genetic gain than strategy GEGV-RND while maintaining a higher genetic diversity at the same time (Figs. 2 and 3). As the selection of parental inbred lines is identical to GEGV-RND, all differences must arise from the cross combination of genotypes. Since only the best allele at each locus is considered, the crossing partners can carry one favorable but up to three unfavorable alleles on one locus, which increases the genetic diversity (Daetwyler et al. 2015), as also shown in the single-index strategy MAXG-TOP. This diversity can then be exploited later by truncation selection.

### Breeding strategies that combine different cross selection indices and/or cross-planning methods

In a third step, we combined different cross selection indices and/or cross-planning methods into breeding strategies, an approach that has not been attempted before. MAXG was the cross selection index that led to the highest genetic gain when combined with truncation selection in the strategies that aimed for maximization of diversity early in the breeding program, without losing any genetic diversity compared to reference strategy GEGV-RND. It was therefore used for at least the cross planning in the generation of the inbred lines in all the breeding strategies that combine two cross-planning methods (Table 3), namely MAXG1/UC1-TOP, MAXG2/UC1-TOP, MAXG2-TOP/UC1-SM, MAXG2-TOP/UC1-SET, MAXG2/UC2-TOP, MAXG2/BPV2-TOP, and MAXG/BPV_ALT-TOP. The next steps were either based on MAXG, the usefulness criterion, or the best progeny value. All combination strategies resulted in a higher genetic gain than the reference strategy GEGV-RND, albeit at different levels (Figs. 2 and 3). The genetic diversity was higher than the reference for most strategies in the oat dataset (Fig. 2). In the wheat dataset, the genetic diversity was higher than in the reference strategy for strategies MAXG2-TOP/UC1-SET and both strategies that combine MAXG with the BPV, either consecutively (strategy MAXG2/BPV2-TOP) or alternating (strategy MAXG/BPV_ALT-TOP) (Fig. 3). We attribute this to the favorable property of MAXG to preserve genetic diversity when applied in early generations. This diversity can then later be exploited by UC-based measures or by the BPV, which led to a higher selection gain than a combination of MAXG in early generations with truncation selection in strategies MAXG1-TOP and MAXG2-TOP (Figs. 2 and 3). The higher genetic diversity that was retained by some of the combined strategies, without compromising genetic gain, also harbors potential for multi-trait selection.

As mentioned, genetic diversity in the context of this study refers to genetic differences in the final DH lines rather than to genetic diversity that is available for later exploitation. This means that if the DH lines have comparable yield but achieve this level with different alleles, they will most likely be different in other traits of interest. Multi-trait selection approaches could therefore benefit from combined breeding strategies even more than single-trait selection because the greater genetic diversity leaves more room for improvement of different traits at the same time.

### Comparison of the two datasets

The simulations with both datasets show that the results are comparable, albeit not exactly identical. Although the oat dataset contains more founder parents and crossing families than the wheat dataset, exotic genotypes were used as resistance donors in wheat, thereby introducing new allele variants and, consequently, additional diversity that is not necessarily pre-selected for desirable traits. The difference between the two datasets is most pronounced for the strategies BPV-TOP and MAXG-TOP which use either BPV or MAXG in all steps. In both cases, these strategies maintain more diversity than the reference strategy RND. However, in the oat dataset, genetic gain was also higher for BPV-TOP and MAXG-TOP than for the reference strategy, whereas in the wheat dataset, the genetic gain was comparable to RND or slightly lower. Both BPV and MAXG are hypothetical values. Their realization depends on the likeliness that there will be recombination between the favorable alleles. For BPV, part of this likeliness is already factored into the measure. This is not the case for MAXG. The pre-selection of alleles for yield by the choice of parents has implications for the performance of the cross-selection indices in the two datasets. When pre-selection can be assumed, it is more likely that the crosses favored by BPV or MAXG will actually show a high yield, even more so for BPV than for MAXG. This is what the results for BPV-TOP and MAXG-TOP reflect (Fig. 2). In the wheat lines, which carry more exotic alleles, the risk that the theoretical value remains largely theoretical is higher. In other words, the possibility that a cross is selected as superior with BPV or MAXG but the resulting offspring carry inferior alleles due to Mendelian sampling and limited recombination is higher in the wheat than in the oat dataset. This manifests itself in the lower genetic gain in wheat compared to oat that is observed in the single-criterion strategies BPV-TOP and MAXG-TOP, in the strategies MAXG1-TOP and MAXG2-TOP, which aim to maximize diversity early, and in the combination strategies MAXG2/BPV2-TOP and MAXG/BPV_ALT-TOP which consist of combinations of BPV and MAXG (Figs. 2 and 3).

### Limitations

For our simulation study, we assumed a purely additive genetic architecture, as we considered line breeding, thereby excluding dominance effects from the calculations. Epistatic effects were not considered because their estimation accuracy is generally low, and no methods currently incorporate epistasis into the cross-planning step rather than in the genomic prediction itself. To increase realism, the breeding scheme used a small family size of ten progeny per cross. Given that each heterozygous genotype occurs only once, this limits the number of crosses that can be made per parent. However, the fixed progeny number did not ensure equal parental contributions, as selection was performed across rather than within families, implicitly allowing unequal parental contributions to the next generation. Optimizing family size was beyond the scope of this study, as it concerns the optimization of the breeding scheme in general (*cf.* Okoye et al. 2026). 

Conclusions from this study are only valid if the simulated results agree with real-life data. In particular, the accurate prediction of the segregation variance that is required for the usefulness criterion remains challenging (Wolfe et al. 2021; Rembe et al. 2022). The low prediction accuracy is attributed to different causes. First, marker effects must be estimated accurately for the predictions to work (Oget-Ebrad et al. 2024). This is a problem not only for the prediction of the usefulness criterion but also for many of the other selection criteria and cross selection indices investigated in this study. However, we hypothesize that the inaccuracies are distributed randomly and therefore do not change the ranking of the breeding strategies relative to each other, with the exception of the usefulness-based breeding strategies because effects are multiplied when the segregation variance is estimated. 

Second, the more quantitative trait loci (QTL) influence a trait, the lower the prediction accuracy for the segregation variance (Oget-Ebrad et al. 2024), which could be problematic for the prediction of yield.

Third, recombination frequencies needed for the estimation of the segregation variance are estimated from the positions of the genetic map. This may cause inaccurcies when the genetic map that is used does not accurately reflect the recombination patterns in the population for which the estimation is carried out.

In addition, estimated recombination frequencies from map positions are generally not very reliable (Kivikoski et al. 2023) and therefore can distort the estimates of the segregation variance.

Fourth, for heterozygous individuals, phased genotypes are required for the estimation of the segregation variance. The phasing step potentially introduces additional noise to the data.

And fifth, the segregation variance is estimated for a progeny population of infinite size. The segregation variance that is actually observed in a limited number of offspring may differ substantially from the estimation (Oget-Ebrad et al. 2024). While the last problem is factored into the simulations, the first four are ignored when marker effects, map positions and phased genotypes are assumed to be known, most likely leading to an over-estimation of the potential of usefulness-based selection strategies. With inaccurately estimated segregation variances, mate allocation will likely become inefficient, leading to a sub-optimal assignment of parents to crosses and subsequently to a lower genetic gain.

The other strategies will be affected by the inaccuracies as well, although to a lesser extent.

In practice, this may lead to differences between the strategies that are smaller than in this simulation study.

However, since the uncertainty is undirected, it should not affect the ranking of the breeding strategies.

The second major limitation of the study is the methodology.

To identify an optimal combination of selection criteria/cross selection indices and cross-planning methods for our breeding scheme, we followed an exploratory approach rather than deriving the combinations from a predefined theoretical principle or formal optimization framework.

Due to the high computational demands of some of the measures employed in the strategies, namely those which estimate segregation variances, it was not possible to conduct an exhaustive grid search of all possible combinations of selection criteria, cross selection indices, and cross planning methods.

Even though estimation of the segregation variance took only fractions of a second per cross for our data, the large number of potential crosses in the different steps of the breeding schem, combined with 300 simulation runs, limited the number of combination strategies that could be evaluated.

Measures were therefore explored in a single-criterion setting first.

Then, the criteria that aim to maximize diversity early in the breeding program were investigated in more detail.

Based on these results, the combination strategies were derived heuristically.

Therefore, while the study addresses the question if there might be potential in using different measures in different stages of the breeding program rather than advocating for one single criterion, there is definitely room for improvement regarding finding the best combinations of measures.

What is considered best will likely depend on the specific breeding program and the specific goals of the breeder.

However, aside from the lack of any specific recommendation, a starting point for breeders trying to optimize their breeding programs might be to consider selection criteria, cross selection indices, and cross-planning methods that go beyond established methods like truncation selection or the usefulness criterion for particular stages of their breeding programs.

The decision should be made depending on the goal of each step of the breeding program, *i.e.* preservation of genetic variation for exploitation in later steps *vs.* realization of as much genetic gain as possible.

If the choice of the measures is made based on this information, it will be possible for breeders to compare a limited number of strategies tailored to their breeding program on a standard desktop computer.

We expect the advantages associated with the specific measures discussed here to be consistent across different datasets, as they arise from properties inherent to the measures themselves. For example, rigid truncation selection based on GEGV is expected to result in relatively high selection gain, accompanied by a corresponding reduction in genetic diversity. Furthermore, we demonstrate that MAXG and BPV differ in their weighting of heterozygous markers/allele frequencies (Figs. 4 and 5), which in turn affects allele fixation. What will certainly change, however, are (1) the magnitude of the effects of the measures on genetic gain and diversity, and (2) the emphasis that is placed on each of the measures’ properties in each specific step of each specific breeding program.

### Conclusions

Our study is the first to combine different selection criteria, cross selection indices, and cross-planning methods into breeding strategies for short-term recurrent genomic selection. Our results show that these combined strategies can result in a higher genetic gain in yield compared to standard truncation selection and random mating of the selected individuals while retaining at least the same amount of genetic diversity at the same time, or, conversely, achieve the same level of genetic gain while retaining more genetic diversity. More genetic diversity means more potential for selection for other agronomic traits and resistances. Combination strategies, therefore, utilize advantages of different criteria and indices and compensate disadvantages. Finding the right combination and order of selection criteria, cross selection indices, and cross-planning methods has the potential to further improve breeding programs but makes optimization even more complex.

## Supplementary Information

Below is the link to the electronic supplementary material.

Supplementary file 1 (pdf 0 KB)

## Data Availability

The analyzed wheat and oat datasets as well as the R scripts can be found in the Github repository [https://github.com/egrosch/short-term-RGS].
