# Supplementary material for: Combining different selection criteria into breeding strategies for short-term recurrent genomic selection
Source: Theor Appl Genet. 2026 Oct 25;139(11):316. doi: 10.1007/s00122-026-05410-7 (PMC13696599; doi:10.1007/s00122-026-05410-7)
Supplement: Supplementary file 1 [file 122_2026_5410_MOESM1_ESM.pdf]

Combining different selection criteria into breeding strategies for short-term recurrent genomic selection

Journal: Theoretical and Applied Genetics

Author names: Emanuel Grosch, Matthias Herrmann, Selma Schurack, Steffen Beuch, Vivek Kurra, Martin Mascher, Raz Avni, Klaus Oldach, Ina Röhrs, Wubishet Bekele, Anja Hanemann, Raja Ram Mehta, Carsten Reinbrecht, Albrecht Serfling, Andreas Stahl, Marco Stucke, Carola Zenke-Philippi

Corresponding author: Emanuel Grosch, emanuel.grosch-1@agrar.uni-giessen.de
Institute of Agronomy and Plant Breeding II, Justus Liebig University, Gießen, Heinrich-Buff-Ring 26-32, 35392, Germany

S1 Calculation of the mean and variance for the UC

The means and standard deviations for a population of heterozygotes derived from two heterozygous parents can be expressed in terms of the additive effects g_j, g'_j of the first and h_j, h'_j of the second parent and the recombination frequencies r_{jk} between two loci j and k. The derivation below is in analogy to the derivations of Osthushenrich et al. (2017) and is mathematically equivalent to the formulations presented by Bonk et al. (2016).

We denote with Z a random variable describing the genotypic value of a heterozygous individual in a family derived from the cross of two heterozygous parents using an additive genetic model. Expectation and variance of Z are

$$\mu = E(Z) = \beta_0 + \sum_c \sum_j E(X_j) + \sum_c \sum_j E(Y_j)$$

and

$$\sigma_g^2 = var(Z) = \sum_c \sum_{j,k} cov(X_j, X_k) + \sum_c \sum_{j,k} cov(Y_j, Y_k)$$

where summation index c is over chromosomes and j, k sums over all locus pairs on a chromosome. The random variable X denotes the genotypic values at the loci on the homologue originating from the gamete of the first parent and Y denotes those on the homologue originating from the gamete of the second parent. The expectations for

locus j are

$$E(X_j) = \frac{1}{2}(g_j + g'_j) \quad and \quad E(Y_j) = \frac{1}{2}(h_j + h'_j)$$

In analogy to the derivations of Osthushenrich et al. (2017)

$$cov(X_j, X_k) = (\frac{1}{2}q_{jk} - \frac{1}{4})(g_j g_k + g'_j g'_k - g_j g'_k - g'_j g_k)$$

and

$$cov(Y_j, Y_k) = (\frac{1}{2}q_{jk} - \frac{1}{4})(h_j h_k + h'_j h'_k - h_j h'_k - h'_j h_k)$$

where

$$q_{jk} = \frac{1}{2} + 2D_{jk}$$

and for gametes of an F1

$$D_{jk} = \frac{(1 - 2r_{jk})}{4}$$

such that

$$q_{jk} = 1 - r_{jk}$$

References

Bonk, S., Reichelt, M., Teuscher, F., Segelke, D., Reinsch, N.: Mendelian sampling covariability of marker effects and genetic values. Genetics Selection Evolution **48**, 1–11 (2016) https://doi.org/10.1186/s12711-016-0214-0

Osthushenrich, T., Frisch, M., Herzog, E.: Genomic selection of crossing partners on basis of the expected mean and variance of their derived lines. PLoS One **12**(12), 0188839 (2017) https://doi.org/10.1371/journal.pone.0188839
